# Investigation of Factors Affecting the Performance of Textronic UHF RFID Transponders

**DOI:** 10.3390/s23249703

**Published:** 2023-12-08

**Authors:** Anna Ziobro, Piotr Jankowski-Mihułowicz, Mariusz Węglarski, Patryk Pyt

**Affiliations:** 1 Doctoral School of the Rzeszów University of Technology, 35-959 Rzeszów, Poland; 2 Department of Electronic and Telecommunications Systems, Rzeszów University of Technology, Wincentego Pola 2, 35-959 Rzeszów, Poland; p.pyt@prz.edu.pl

**Keywords:** RFID transponder, smart textiles, smart fabrics, wearable devices, textile antennas, wearables, e-textiles, RFIDtex tag

## Abstract

The aim of this paper is to demonstrate progress in textronic UHF RFID transponder (RFIDtex tag) technology. The fundamental idea behind the RFIDtex tag design involves galvanic separation between circuits of the sewn antenna and the chip, which are electromagnetically coupled through a system of inductive loops. To advance the development of this concept, it is crucial to detect factors affecting the performance of the transponders. To achieve this goal, a mathematical model of the textronic UHF RFID transponder was developed. It involves relationships that describe the impedance of each element, the mutual inductance of the loops, and the chip voltage, and it enables the exploration of the influence of these variables on general parameters such as impedance matching and read range. Various analytical and numerical approaches were considered to obtain the value of the mutual inductance of the loops. The dimensions and geometry of the antenna, as well as the matching circuit in the microelectronic module, were taken into account. Based on the mathematical model, it was determined that mutual inductance strongly affects the chip voltage for frequencies higher than 800 MHz. The calculations from the mathematical model were compared with numerical simulations. Experimental studies were also conducted to investigate how the transponder performance is affected by either the distance between the centers of the loops or the conductivity of the threads used to embroider the antenna. The measurement results allowed us to conclude that even small imperfections in the manufacturing of the transponder, which slightly increase the vertical or horizontal distance between the centers of the loops, cause a dramatic decrease in the mutual inductance and coupling coefficient, significantly impacting the transponder’s performance.

## 1. Introduction

### 1.1. Textronic UHF RFID Transponders

In recent years, numerous research studies have addressed RFID wearables, mostly due to the expansion of the Internet of Things, as microwave and wireless systems are becoming eligible and ubiquitous in everyday life [1]. Among the developed solutions, a large portion pertains to medical and healthcare devices, such as sensors for measuring vital signals [1,2,3]. Other examples of RFID applications include positioning, localization, and tracking human activities [4,5]. Textile-based RFID tags need to meet various requirements like flexibility and low production costs, as well as challenges posed by mechanical stress, washing processes, or operation disruptions caused by proximity to the human body [6,7]. To achieve these goals, various conductive materials and technologies for their application are being investigated, such as nanocomposites, carbon nanotubes [8], conductive pastes, inks, fibers, or fabrics [9,10,11,12,13]. For example, according to the reference literature, graphene is used as a conductor due to its good mechanical and electrical properties. Its implementation in RFIDtex tag designs provides the opportunity to achieve a highly conductive, flexible, and mechanically robust material. Numerous examples of research studies in this area include conductive pastes [14], graphene sheets [15], laser-based methods such as laser-induced graphene [16,17,18], flexible graphene assembly films [19,20,21], or printing techniques [3,22,23,24]. However, the development and widespread use of these technologies encounter challenges, such as toxicity to humans and the environment, along with the need to implement a costly and complicated manufacturing process that is not commonly used or known in the textile industry [25]. Also, washability limitations of such experimental materials can be observed [26].

However, antennas in RFIDtex tags are frequently made by embroidering or sewing conductive threads [27,28,29,30,31,32,33,34,35,36], by knitting [37], or using any other method known in the textile industry. These proposals differ in their used threads, substrates, antenna structures, and types of integrated impedance matching elements. There are a wide variety of antenna types designed by researchers, including dipole, slotted patch, two symmetrical circular patches, meander line, fractal, rectangular or octagonal geometry, text-meandered, or even Mickey Mouse shaped. In the literature, alongside descriptions of works on specific solutions, there is also research aimed at solving problems that occur when embroidering, sewing, etc., like yarns deformation, accuracy of project reproduction, influence of machine settings, etc. [38,39,40]. Other papers address methods for determining the conductivity of embroidered antennas [35] or the use of polymers in conductive fibers [30,41], which are a desirable material in wearable electronics due to their flexibility [42,43,44].

Instead of using conductive fibers, other conductive flat materials can be employed in tag structures [45,46,47,48]. Such tags can be attached to the textile product using glue and ironing. In paper [48], a tag with a split-ring resonator antenna is proposed. It consists of two layers of conductive fabric. Nevertheless, other examples of UHF RFID tags made using commercially available conductive ink and the printing method can be found in references [12,49].

Among the solutions of wearable UHF RFID tags, there are also those that serve as sensors, including strain sensors [45], handwriting sensors [50], and surface crack monitoring [51]. On the other hand, the review in reference [52] addresses issues of e-textile UHF RFID tag design methodology, committed to overcoming problems related to interferences with their operation.

A particular problem for e-textile RFID transponders is the connection between the chip and the antenna. Efforts are undertaken to find ways to replace soldering, which has a temperature unsuitable for most textile materials [53]. One way is to use low-temperature soldering [26]. However, the most common way is applying bonding with conductive epoxy [27,31,35,47,48]. Also, examples of knitted [37] and embroidered interconnection can be found [29,54]. In paper [53], a comparison of three different methods of connection, sewing, snap buttons, and inserting, is presented. Also, a proposal for chipless embroidered RFID tags is found in reference [30]. Based on what has been presented so far, the work in reference [28] stands out, in that there is no physical connection between the antenna and the chip. The transponder consists of a linear dipole antenna and a rectangular coupling circuit of the chip. Both parts are embroidered and galvanically separated but inductively coupled. Changes in coupling between these components are used to measure displacement and strain.

This tag is similar to the textronic UHF RFID transponder [55], where the antenna and chip are inductively coupled instead of using a galvanic junction. This transponder differs from that in reference [28] in the construction of the antenna, where the coupling element is placed in the form of a loop, the method of fabricating the chip coupling circuit is different, and there is the application of both a linear dipole and a meander line antenna.

### 1.2. Aim of Research

This paper addresses issues related to textronic UHF RFID transponders which are integrated into fabrics. This idea is unique in terms of the galvanic separation between the transponder antenna and the chip. Due to the inductive coupling between these components, the RFIDtex tag construction includes two additional elements: a coupling circuit of the antenna and a coupling circuit of the chip. The antenna coupling circuit is fabricated using the same method as the antenna, whereas the chip coupling circuit, along with the galvanically connected chip, is made as a typical microelectronic device. Therefore, the concept envisions their production as separate modules: an antenna module and a microelectronic module. Both parts may be hidden in clothing, or they may also take the form of a decorative or functional garment element, such as a button, bead, emblem, etc.

**Figure 1:**
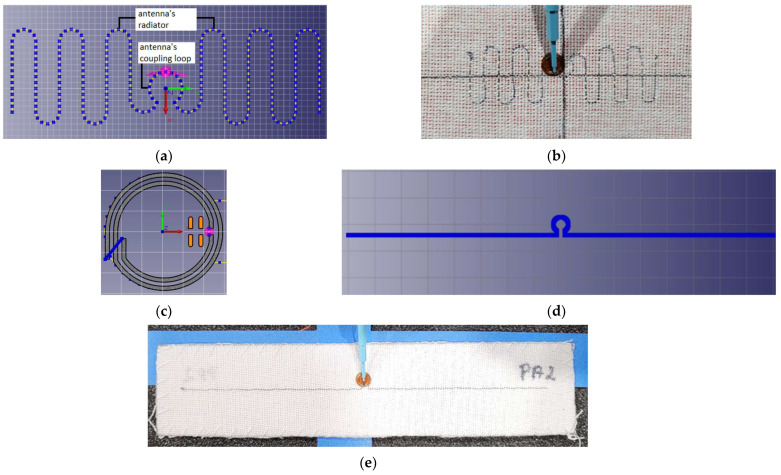
Textronic UHF RFID transponders: (**a**) EMCoS Studio simulation model of meander line antenna; (**b**) real sample of RFIDtex tag with meander antenna; (**c**) simulation model of chip coupling circuit; (**d**) EMCoS Studio simulation model of dipole antenna; (**e**) real sample of RFIDtex tag with dipole antenna.

In developing this concept, emphasis is placed on utilizing manufacturing techniques that are most widely used in the textile industry. For this reason, the antenna module is sewn or embroidered using conductive threads. Nevertheless, this concept allows for the use of other suitable materials and methods. The microelectronic module is manufactured separately and then distributed to clothing factories, where it can be easily sewn or attached to fabric. The numerical model of the textronic UHF RFID transponder’s components and fabricated samples of two constructions that differ in the antenna type are presented in Figure 1. The microelectronic module is the same in both cases.

The aim of the conducted research is to present factors impacting the performance of textronic UHF transponders. The main factors influencing the antenna impedance and chip voltage were determined. Among them were mutual inductance between the antenna and the chip coupling circuits and the thread resistivity. The simulation was conducted to establish its impact on the chip voltage and antenna impedance. Then, experimental research on the impact of the distance between coupling circuits, affecting mutual inductance, and embroidered antenna resistance on transponder antenna impedance and read range was performed.

The major issue in the further development of this concept is the lack of knowledge regarding crucial factors affecting the effectiveness and performance of the textronic UHF RFID transponder, their importance, and the relationships between them. The aim of this study is to identify these factors and to determine their impact on the transponder’s operational parameters. The only research that has addressed this topic involves experimental studies on the influence of textile substrates [56] and the washing process [57]. Our investigation is the first attempt at a mathematical description of the textronic UHF RFID transponder’s performance. The results obtained will allow us to define rules for designing and manufacturing properly working transponders according to specific application areas and required operational parameters. Likewise, they will shed light on development opportunities.

Development directions include both improving effectiveness and designing new transponder constructions that meet the condition of inductive coupling between the antenna and chip. The existence of various constructions of the textronic UHF RFID transponders is necessary in view of the nature of textiles and the clothing industry. The antenna geometry and microelectronic module shape are determined not only by their required performance but also by the available space on the fabric, its composition and type, the product designation, and aesthetics. The constructions considered in this paper consist of microelectronic modules manufactured as round elements like buttons or sequins attached plainly to fabric. However, such components are not used in all kinds of clothes.

The next interesting direction is the estimation of the RFIDtex tag’s usefulness in automatic identification systems beyond the production and sales stages, including in Internet of Things implementations or as wearable RFID sensors. These application areas involve understanding transponder performance and how operational parameters change due to product use, integration, and destruction, as well as the impact of environmental conditions and the human body. It is presumed that the development and dissemination of this kind of wearable transponder could help advance the expansion of RFID systems in the textile industry and IoT, exploited at each step of production, sale, use, waste disposal, and recycling of textile products.

## 2. Materials and Methods

Firstly, mathematical calculations based on common electromagnetic laws were made in order to determine the relationships between the basic parameters of the transponder. The mathematical model was used to derive the formulas for antenna impedance, chip voltage, and mutual inductance between the coils of the coupling system. These expressions allowed us to obtain theoretical knowledge on the changes in electrical parameters depending on various factors. The conducted investigations led to the identification of research directions and the verification of laboratory measurements.

MATLAB was used to plot graphs illustrating the relationships between the considered factors based on the obtained relationships. In previous work on concept development, simulations were carried out using the EMCoS Studio 2022 software [55,56,57]. These outcomes were supplemented with measurements of magnetic field parameters. The next stage of the investigation involved experimental studies on the influence of two performance factors detected in the previous stage: the distance between the centers of the coupling circuits (both vertical and horizontal) and the embroidered antenna resistance.

**Figure 2:**
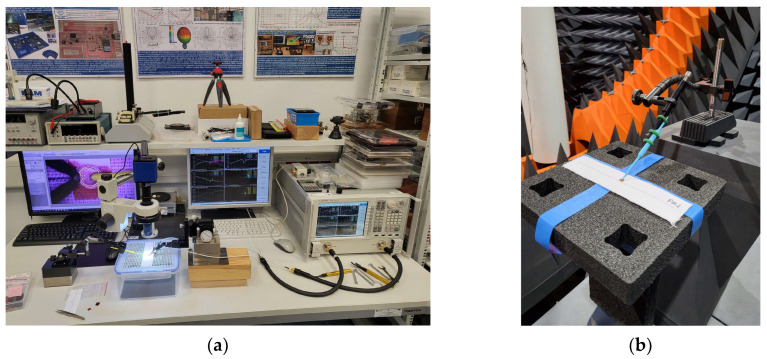
Laboratory research stand: (**a**) stand for impedance measurements; (**b**) stand in anechoic chamber.

The samples were sewn using an embroidery machine, BROTHER INNOV-IS V3. The maximum rotational speed of the embroidery machine’s bobbin was 350 rpm. The tension of the upper thread (non-conductive), supporting the lower conductive thread, varied depending on the thickness and stiffness of the conductive thread. The bobbin also had an adjustable screw to regulate the tension of the thread, but this feature was not used during embroidery. The antenna impedance measurements were conducted using a Keysight PNA-X N5242A vector network analyzer, PacketMicro DPSS201505 SS05–0053 probe, and microscope to verify proper contact between the probe and the circuit. The read range measurements were performed in a Microwave Vision Group anechoic chamber equipped with a Voyantic Tagformance Pro measurement system with Tagformance UHF v.13.2.3 software (Figure 2). 

A epsilometer system with a Compass Technology measuring device and Vector Network Analyzer (VNA) Copper Mountain R60 was used to obtain the dielectric permittivity of the textile substrates.

## 3. Results

### 3.1. Calculations and Simulations

#### 3.1.1. Mathematical Model of Textronic UHF RFID Transponder

**Figure 3:**
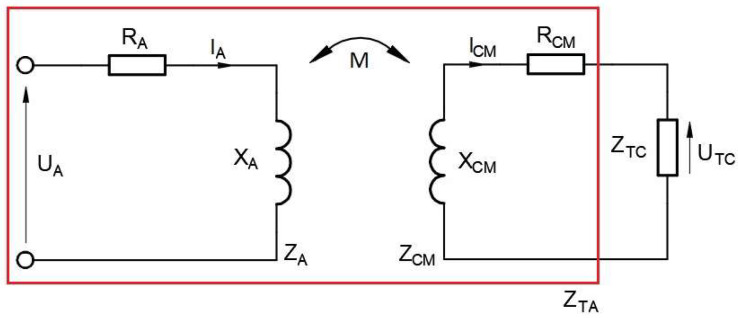
Electrical equivalent of the radiofrequency circuit in the textronic UHF RFID transponder.

The first stage of the investigation involved a mathematical description of the textronic UHF RFID transponder based on common electrical and electromagnetic laws. The research purpose was to determine the relationship between the complex impedance of the transponder components and the chip voltage. Both of these parameters refer directly to the operational parameters, such as impedance matching and the read range. The electrical equivalent of the radiofrequency frontend in the RFIDtex tag is presented in Figure 3.

The left part of the circuit diagram corresponds to the antenna module comprising the embroidered antenna and the coupling coil, where the impedance *Z_A_* consists of the resistance *R_A_* and reactance *X_A_*:

(1) $$Z_{A}=R_{A}+jX_{A}$$

The *U_A_* value represents the voltage induced in the antenna module, and *I_A_* is its current.

The second component of the transponder, the microelectronic module, forms the right circuit. It is combined with the chip, which has impedance *Z_TC_*, and the coupling coil, whose impedance *Z_CM_* is expressed as:

(2) $$Z_{CM}=R_{CM}+jX_{CM}$$

The inductive coupling between both modules is described by mutual inductance *M*. Although the antenna module is sewn on fabric, the proper transponder antenna also includes a part of the microelectronic module. It is a combination of the left circuit described by *Z_A_* and the chip coupling circuit (*Z_CM_*) joined inductively, as indicated by the red line in the diagram. The impedance value of these components is required to determine the impedance matching between the chip and the transponder antenna.

Since the electric circuit diagram of the RFIDtex transponder (Figure 3) is similar to that of an air-core transformer, an equivalent circuit of a two-port network was created to obtain the desired relationships. The impedance *Z_M_* in the equivalent circuit was expressed as an impedance consisting solely of the imaginary part *X_M_*:

(3) $$Z_{M}=jX_{M}$$

The following relationship for the antenna impedance was determined:

(4) $$Z_{TA}=Z_{CM}+\frac{X_{M}^{2}}{Z_{A}}$$

Using the same equivalent circuit, the chip voltage *U_TC_* can be obtained:

(5) $$U_{TC}=I_{CM}\cdot Z_{TC}=\frac{U_{A}\cdot jX_{M}\cdot Z_{TC}}{Z_{A}\left(Z_{TC}+Z_{CM}\right)+X_{M}^{2}}$$

where *I_CM_* is the current generated in the chip coupling circuit. The chip voltage is crucial in determining the power delivered to the chip. It influences the read range in the RFID system. For this reason, it is desirable to design RFIDtex transponders in a way that allows the generation of higher current in the microelectronic module than in the others.

In the procedure of obtaining the impedance *Z_TA_* or the voltage *U_TC_*, the main problem is to determine the value of *X_M_*. In the case of the antenna impedance, the dependency on *X_M_* is quadratic, but in the case of the chip voltage, the function exhibits a maximum. Attempts to obtain the optimal value of mutual inductance between coupling circuits will be the focus of future research. In the present investigations, various approaches to determine the value of *X_M_* were taken into consideration, as described in the next section.

#### 3.1.2. Mutual Inductance between Coupling Circuits

The mathematical model of the textronic UHF RFID transponder is a significant tool for addressing measurement issues related to its parameters. In the laboratory, only the impedance of the transponder antenna and the impedance of the chip coupling circuit need to be measured. Although the impedance of the embroidered antenna can be obtained as a result of numerical model computations in EMCoS Studio, there is no software feature to directly compute the impedance of mutual inductance.

The first approach to determining the value of *X_M_* is based on the developed mathematical model. Equation (4) is rearranged into the following form:

(6) $$X_{M}=\sqrt{\left(Z_{TA}-Z_{CM}\right)Z_{A}}$$

The numerical values of the impedances used for further calculations can be obtained from numerical computations or laboratory measurements. In the presented research, only numerical computations are used. The limitation of this method arises from the accuracy of measurements and software simulations. However, its main advantages will become apparent when compared to the next two approaches, which use analytical electromagnetic formulas. These formulas are useful solely for simple geometries of the coupling system and only under certain assumptions.

In the investigations, two constructions of the RFIDtex transponder were considered: the first one with a 16 cm long dipole antenna and the second one with a meander line antenna. Both constructions have the same antenna and chip coupling circuits. The antenna coupling coil is a loop with a radius of 2.85 mm sewn in the middle of the radiator. The chip coupling coil consists of two connected concentric loops, with the external loop having a radius of 2.85 mm and the internal loop having a radius of 2.45 mm. In this basic case, the mutual inductance can be obtained through calculations based on common analytical formulas of electromagnetic laws. The first step is obtaining the magnetic flux density *B* according to the Biot–Savart law for a circular loop of radius *r* carrying a current *i*, expressed as [58]:

(7) $$B=\frac{\mu_{0}i}{2r}$$

It is assumed that the magnetic flux density is the same across the entire area of the loop and equals the value occurring at the center. That formula does not include the impact of the magnetic field generated by the arms of the antenna. Next, the magnetic flux is determined for the circular area of the second circuit as follows [59]:

(8) $$\Phi_{12}=\overset{}{\underset{S}{\iint }}BdS=\frac{\mu_{0}i}{2}\pi r$$

And finally, the mutual inductance *M* is obtained [59]:

(9) $$M_{12}=\frac{\Phi_{12}}{i}=\frac{\mu_{0}}{2}\pi r$$

To calculate *X_M_*, it was assumed that reactance is proportional to the frequency:

(10) $$X_{M}=j2\pi fM$$

**Figure 4:**
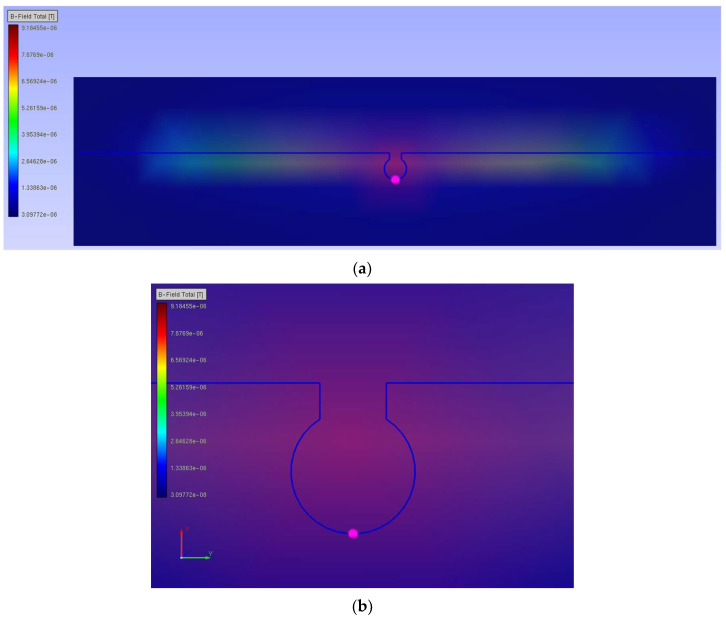
Magnetic flux density of 16 cm long dipole antenna for 860 MHz: (**a**) coupling circuit and radiator; (**b**) coupling circuit.

The third method was implemented to avoid some of these assumptions. It uses EMCoS Studio features to obtain some parameters of the magnetic field in the RFIDtex transponder components. The simulation was performed with *U_A_* = 1 V. The magnetic flux density for the 16 cm long dipole antenna is presented in Figure 4.

**Figure 5:**
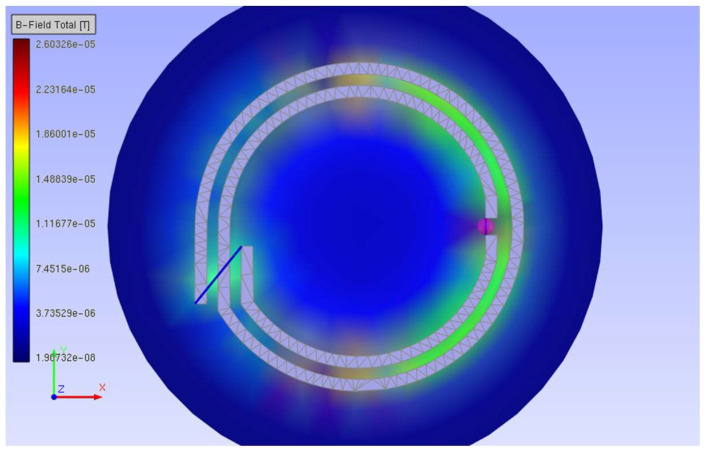
Magnetic flux density of chip coupling circuit for 860 MHz.

The magnetic flux density of the chip coupling coil is shown in Figure 5.

**Figure 6:**
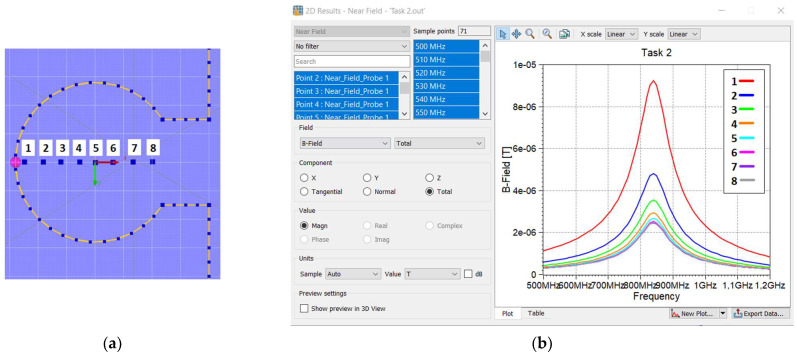
Computation of magnetic flux density inside loop of coupling circuit: (**a**) location of measurement points; (**b**) results.

From the graphs, it can be seen that *B* is not constant throughout the area, and for the chip coupling circuit, its value is highly diversified between the center and the edges. The use of a near field probe allows us to obtain the magnetic flux density at the selected points. The points are located on the diameter of the loop (Figure 6a), and the *B* value is obtained for each of them (Figure 6b).

**Figure 7:**
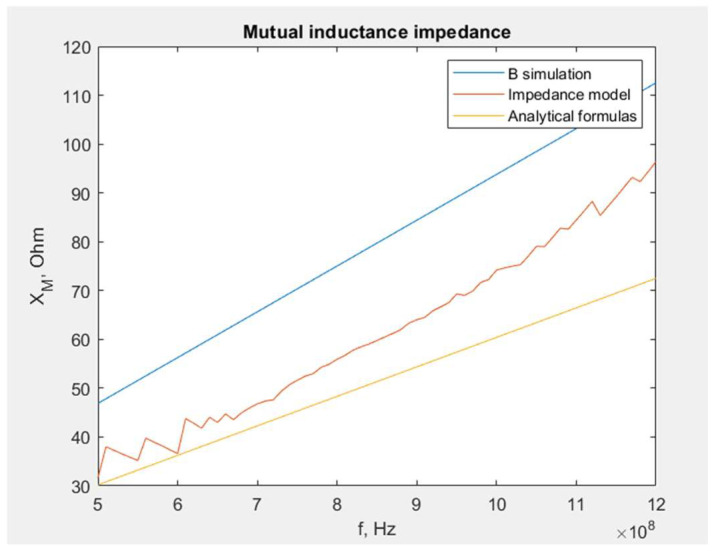
Comparison of different approaches used to obtain the *X_M_* value for a 16 cm long dipole antenna.

The highest value occurs at the point closest to the edge. In the third approach, this highest *B* value is used instead of analytical formula (7) to obtain the magnetic flux density. In this way, the drawback of previous approach is eliminated, where the calculated value of *B* is assumed in the center of the loop. As can be seen in Figure 7, this value is significantly lower at this point. Equation (8) is also used, but in (9), the embroidered antenna current *I_A_* value is required. In the investigation, it is calculated from mathematical model relationships:

(11) $$I_{A}=\frac{U_{A}\left(Z_{TC}+Z_{CM}\right)}{Z_{A}\left(Z_{TC}+Z_{CM}\right)+X_{M}^{2}}$$

but it could also be computed by EMCoS Studio software.

The mutual reactance *X_M_* obtained on the basis of the developed methods is presented in Figure 7.

As can be presumed based on the derived assumptions, the *X_M_* value derived from the analytical formulas, assuming a smaller *B* value at the center, is the lowest one. Similarly, the approach with a higher *B* value at the edge gives excessive results. The values determined by the mathematical impedance model are located between them. From this comparison, the following conclusion may be drawn: using a mathematical model is a proper and reliable approach to obtain the *X_M_* value. Other methods could also be used to approximate *X_M_*, but only for simple circuit geometry.

**Figure 8:**
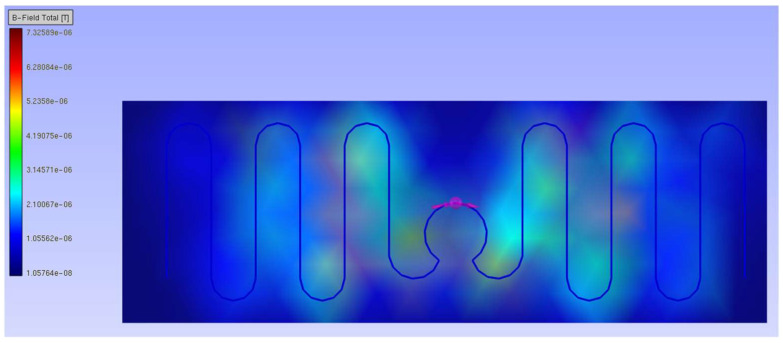
Magnetic flux density of the meander line antenna.

The second design with the meander line antenna is an example of a more complex geometry, where methods based on the analytical description of electromagnetic parameters fail due to their assumptions. Although the coupling circuit is the same as before, its area is interfered with by the magnetic field derived from the antenna arms (Figure 8).

**Figure 9:**
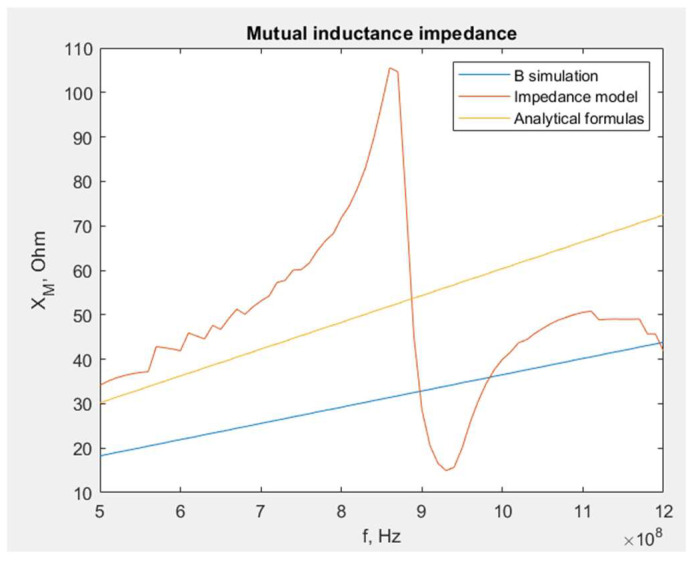
Comparison of different approaches used to obtain the *X_M_* value for the meander line antenna.

The same steps for the dipole antenna may be followed. The influence of the magnetic field generated by the antenna arms is noticed solely using the mathematical model (Figure 9).

For these reasons, in the next steps of this research, the assumed *X_M_* value for each construction is that obtained by the mathematical model.

**Figure 10:**
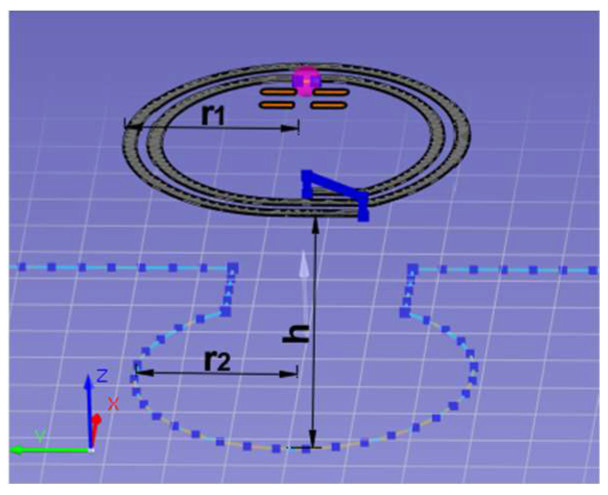
Geometry of coupling circuits.

Another measure of magnetic coupling is the coupling coefficient, which qualitatively describes the coupling between circuits [60]. When the loops are centered on the same axis z, it can be determined as:

(12) $$k\left(h\right)=\frac{r_{1}^{2}\cdot r_{2}^{2}}{\sqrt{r_{1}\cdot r_{2}}\cdot {\sqrt{h^{2}+r_{1}^{2}}}^{3}}$$

where *h* is the distance between loops, as illustrated in Figure 10.

The coupling coefficient takes values from 0 to 1, where 0 means no coupling and 1 represents total coupling, assuming that the same magnetic field is passing through an area of both loops.

**Figure 11:**
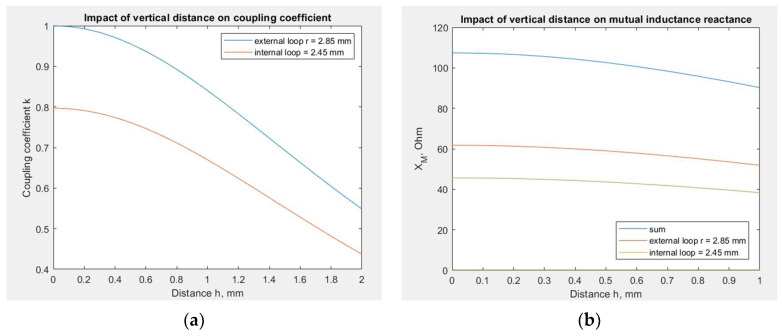
Impact of vertical distance on: (**a**) the coupling coefficient; (**b**) the reactance *X_M_* of the RFIDtex transponder with a meander line antenna.

Equation (12) can be applied to approximate the impact of the vertical distance between the coupling circuits. This relationship is crucial for the RFIDtex transponder, especially due to possible failures in its manufacturing or the displacement of the fabric from the microelectronic module during garment use. For example, an elastic fabric with embroidery hung on a hanger may no longer adhere to the rigid button with the chip and its coupling circuit. As seen in Figure 11a, the coupling coefficient decreases dramatically at a distance of 2 mm; therefore, the RFIDtex transponder cannot work in the case of the above example. 

The influence of the coupling coefficient on the *X_M_* reactance for a shorter distance of 1 mm in the transponder with the meander line antenna is shown in Figure 11b. The value of *X_M_* for no distance is obtained from the mathematical model for a frequency of 866 MHz and equals 107 Ω. With increasing distance, the decrease in the *X_M_* value becomes greater.

**Figure 12:**
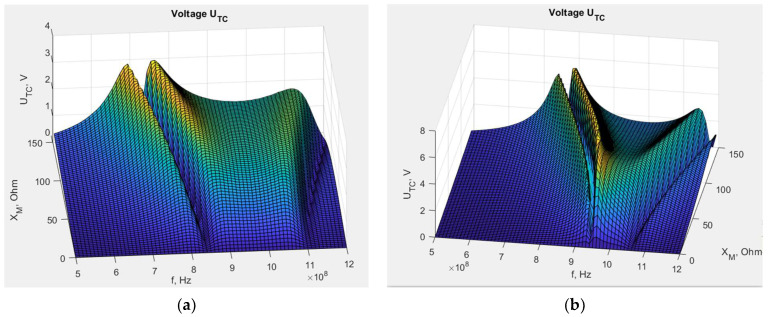
Chip voltage dependency on reactance *X_M_*: (**a**) transponder with dipole antenna; (**b**) transponder with meander line antenna.

The mathematical model, combined with EMCoS Studio computations, allows us to simulate the impact of changing the *X_M_* reactance on the chip voltage *U_TC_*. The results obtained on the basis of Equation (5) for the dipole and the meander line antenna are presented in Figure 12. The impedance values are obtained from numerical computations, the chip impedance *Z_TC_* equals 15.3—j313 (on the basis of Ucode 7m SL3S1214 datasheets by NXP Semiconductors), *U_A_* is assumed to be 1 V, and *X_M_* varies from 0 to 150 Ω based on the values obtained in the previous stage. 

This large frequency bandwidth is displayed in the presented graphs because, at this stage of research, we were interested in observing the behavior of the *U_TC_* function with changes in the selected variables, as well as identifying the position of its local and global maxima and minima. This knowledge is essential for investigating constructions of this type with inductively connected modules and for understanding the phenomena occurring within them. Only after completing this step it is possible to analyze the posed issues in the context of practical application in RFID technology. The ultimate goal of this study is to find the maximum *U_TC_*. As can be observed, the voltage varies significantly with frequency, and for certain values of *X_M_*, the maximum *U_TC_* values are not achieved in the frequency band specific for RFID systems. Furthermore, at this stage of the research, a method for determining the geometric dimensions of the transponder elements to achieve resonance has not yet been developed. For this reason, it is beneficial in analyzing the results of transponder simulations and measurements, both current and future, to have calculations for a broader frequency range. Of course, in the future, the investigated frequency range will be gradually limited until only standard frequencies become the subject of interest.

The different shapes of the graphs show that the antenna geometry has a significant impact on the chip voltage, and consequently, on the read range. For the meander line antenna, the maximum voltage is higher than for the dipole, but in that case, these maximal values dramatically decrease with variations in the *X_M_* reactance. This stronger dependency can indicate that the meander line antenna is less stable in the case of unintentional changes in its construction, such as displacement of the microelectronic module relative to the antenna coupling circuit, and its performance may significantly degenerate. This implies better performance but also greater vulnerability to manufacturing inaccuracies or usage destructions. However, these conclusions are valid only for certain values of *X_M_*, because, as seen in Figure 12, there are regions of milder and stronger changes. Not necessarily for every value of *X_M_*, the region of strong changes for the meandered antenna corresponds to the region of milder changes for the dipole antenna.

From the viewpoint of further development of these constructions, it can be stated that the tag with a dipole antenna and *X_M_* = 63 Ω at frequency 866 MHz is far from its optimal point, and there is a need to improve the efficiency of its coupling circuit. In contrast, the meander line antenna with *X_M_* = 107 Ω at frequency 866 MHz is close to the optimal point, and its *X_M_* value is excessive. In this case, the deterioration of the coupling circuit efficiency may cause performance improvement. However, considering the assumptions made regarding calculations and simulations, the obtained numerical values are approximated and may lead to incorrect conclusions about the search for the optimal value for a given construction. This issue will be investigated in the future.

**Figure 13:**
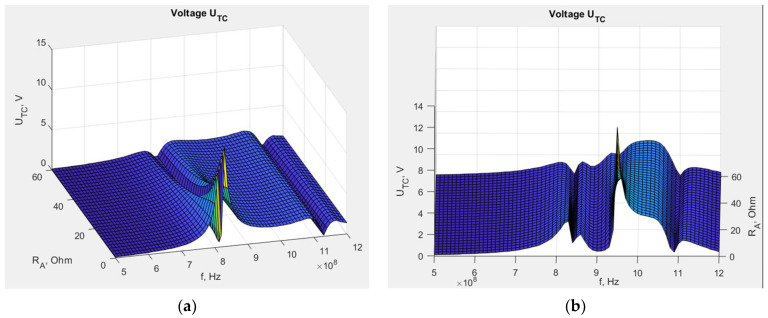
Chip voltage dependency on embroidered antenna resistance: (**a**) transponder with dipole antenna; (**b**) transponder with meander line antenna.

The next factor affecting the RFIDtex transponder is the resistivity of threads used in the antenna embroidering. The real part of the embroidered antenna impedance can be calculated based on its length and thread resistivity. The relationship between the chip voltage and the antenna resistance can be obtained using Equation (5) and the same numerical computations (Figure 13).

The graph shape also depends on the antenna geometry. The significant influence of resistance is observed for the dipole antenna in the range of 800–900 MHz, where the chip voltage decreases with increasing *R_A_*. However, at the same time, the decrease itself becomes smaller until a certain value of *R_A_*, and then the value stabilizes. For the meander line antenna, the same phenomenon occurs for the band around 970 MHz. For other frequencies, *R_A_* has no great impact on *U_TC_*.

**Figure 14:**
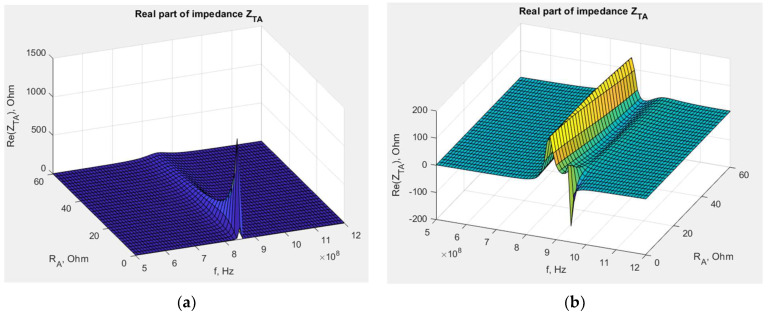
Real part of transponder antenna impedance dependency on embroidered antenna resistance: (**a**) transponder with dipole antenna; (**b**) transponder with meander line antenna.

**Figure 15:**
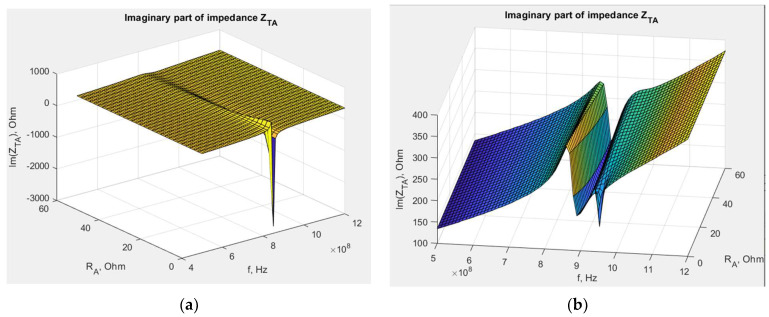
Imaginary part of transponder antenna impedance dependency on embroidered antenna resistance: (**a**) transponder with dipole antenna; (**b**) transponder meander line antenna.

Equation (4) is used to obtain the dependency of transponder antenna impedance *Z_TA_* on the embroidered antenna resistance *R_A_* (Figure 14 and Figure 15).

The influence of the antenna geometry is also crucial. The real part of transponder impedance decreases for the dipole antenna, and later, it is constant at increasing *R_A_* values. However, this dependency is more complex for the meander line antenna, and a small or no impact is also observed at most frequency values. 

### 3.2. Experimental Research

#### 3.2.1. Impact of Vertical Distance between Antenna and Chip Coupling Circuits

For the experimental investigation, the meander line antenna analyzed in Section 3.1 was used. It was sewn using Agsis Syscom thread with a global thread tension of 0 and a tension of 9. The distance between the coils in the coupling system was increasing by inserting pieces of Kapton between them. Each piece was 125 µm thick.

**Figure 16:**
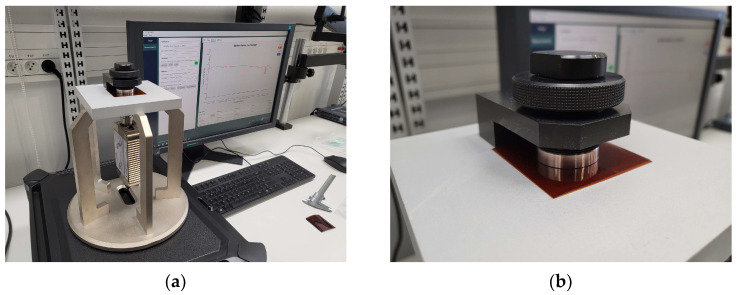
Laboratory research stand: (**a**) epsilometer; (**b**) piece of Kapton under testing conditions.

The first step of the investigation involved measuring the dielectric permittivity of Kapton using the epsilometer (Figure 16).

**Figure 17:**
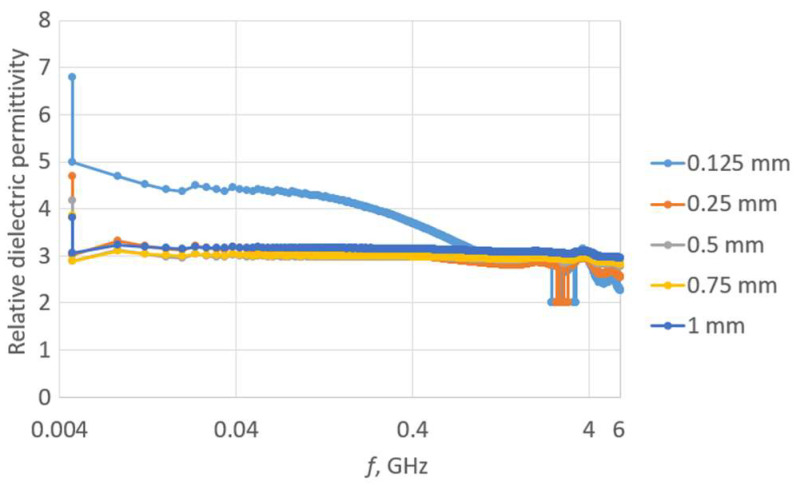
Relative dielectric permittivity of Kapton spacers.

The measurement was repeated for each subsequent piece of Kapton placed on the remaining stack. This way, spacers with thicknesses of 125, 250, 500, 750, and 1000 µm were achieved. The relative dielectric permittivity for each separator is presented in Figure 17.

**Figure 18:**
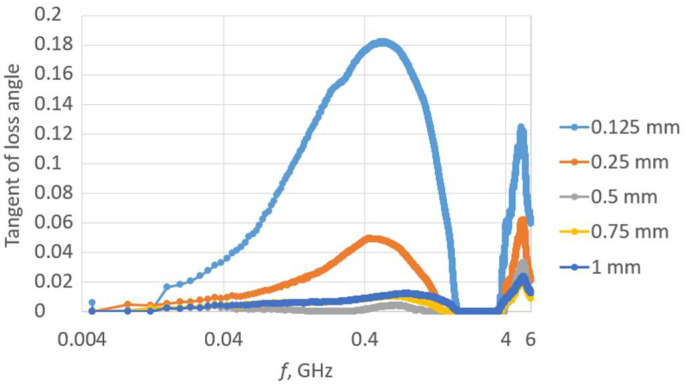
Tangent of the loss angle of Kapton separators.

Except for the first case for the single Kapton layer, the relative dielectric permittivity was similar for subsequently increasing spacers. The tangent of the loss angle for each separator is shown in Figure 18. As the thickness increases, smaller changes in the tangent of the loss angle are observed.

**Figure 19:**
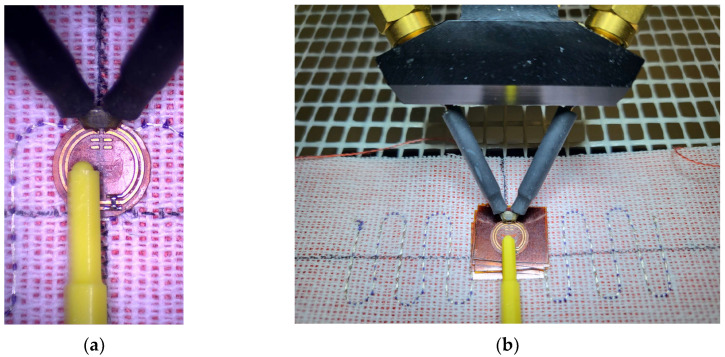
Measurements of vertical distance impact: (**a**) coupling circuits exactly aligned with no vertical or horizontal distance; (**b**) coupling circuits with a vertical separator.

**Figure 20:**
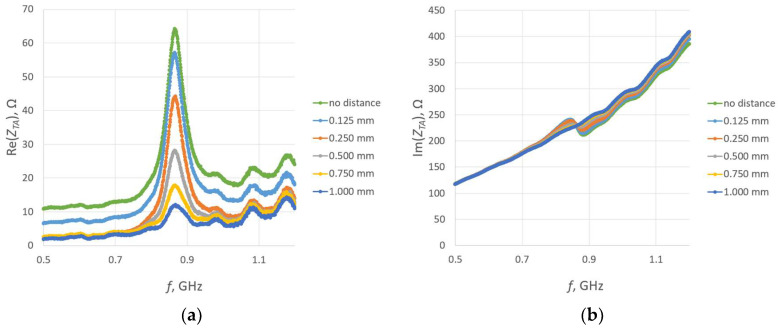
Impedance of transponder antennas *Z_TA_*: (**a**) real part *R_TA_*; (**b**) imaginary part *X_TA_*.

In the next experiment, the transponder antenna impedance measurements were conducted for each separator and with no distance between the coupling circuits (without any Kapton layer). The test setups are shown in Figure 19, and the results are shown in Figure 20.

The relationship between the vertical distance and the real part of the impedance is consistent with the numerical calculations shown in Figure 11. The larger the distance between the coupling circuits, the lower the magnitude in the real part of the impedance. Also, the magnitude of decrease is lower. The impact on the imaginary part of impedance is also visible. With increasing distance, the resonance becomes smaller, until it eventually disappears.

**Figure 21:**
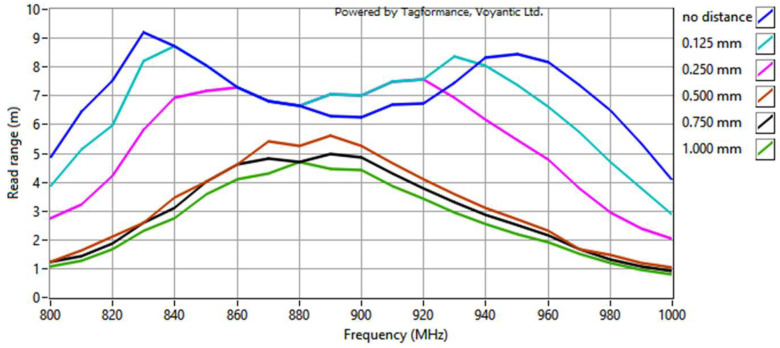
Read range of RFIDtex transponders with a vertical distance between the microelectronic module circuit and the antenna coupling circuit.

The final stage involved measurements of the read range (Figure 21). The parameters of the Voyantic sweep settings were as follows: minimum power of −5 dBm, maximum power of 25 dBm with a step of 1 dB, start frequency of 800 MHz, and stop frequency of 1000 MHz with a step of 10 MHz. The sweep direction was rising. The transmitter had an output power of 29 dBm, and its antenna had a maximum gain of 6 dBi. The sensitivity of the receiver was −70 dBm, and its antenna had a 6 dBi gain. The ISO 18000-6C communication protocol was applied with the query command. The forward link was 25 µs DSB-ASK, and the return link was FM0, 40 kHz. The wideband UHF reference Tag v1 delivered with the Voyantic system was used to set up the measurements.

The shape of the waveforms obtained for the separator cases: no distance, 0.125 mm and 0.250 mm, is consistent with the surface of the graph for *U_TC_* dependency in Figure 12b. The different shapes of the remaining curves may result from the attenuation of the magnetic field influence generated by the meander line antenna with increasing distance. In consequence, the obtained results are more similar to the surface of the respective graph for the dipole antenna (Figure 12a), and the decrease is not as dramatic as for the meander line antenna or the read range for smaller distances. The disappearance of the two extremes is also noticeable with the decrease in *X_M_* related to the increase in the distance.

In light of the earlier considerations, the decrease in *X_M_* should result in better transponder performance, because the optimal value is lower than achieved by this construction. However, this phenomenon is not observed. This could have happened due to the assumptions made in the calculations, causing the obtained values to be approximate, or due to the loss of the original geometry as the circuits moved away from each other. This issue must be further investigated in the future.

#### 3.2.2. Impact of Horizontal Distance between Antenna and Chip Coupling Circuits

During manufacturing or use of clothes, horizontal displacements of the circuits can also occur. Such displacements cause variations in the coupling coefficient and mutual inductance as a result of changes in the common surface area of the coupling coils and limitations in the magnetic flux.

**Figure 22:**
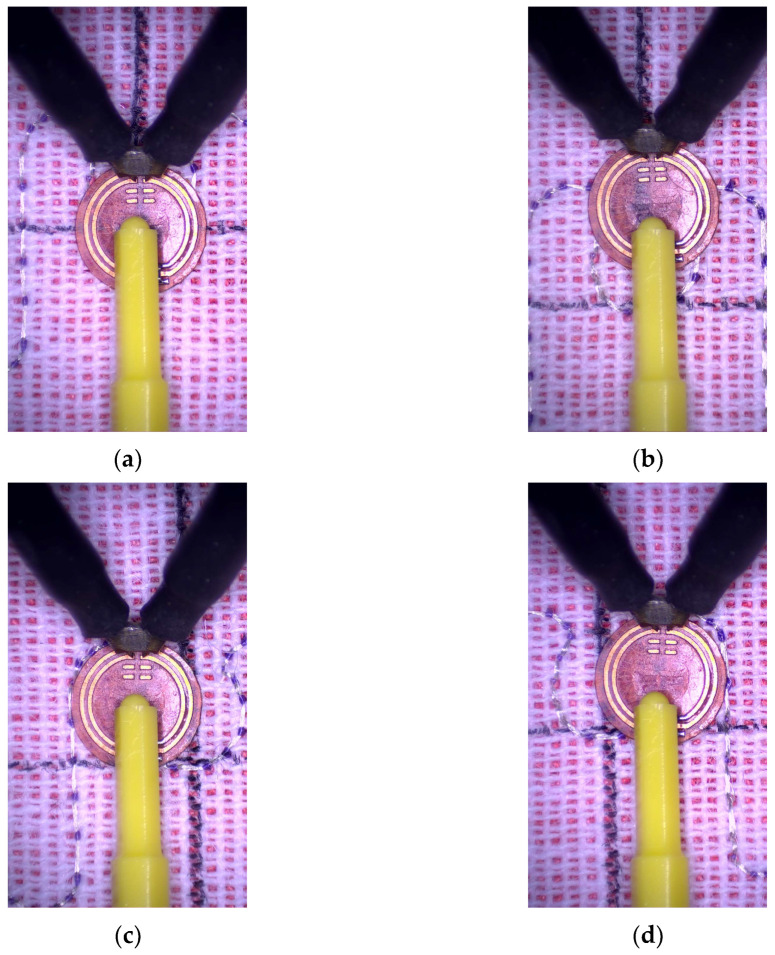
Location of chip coupling circuit relative to antenna coupling circuit: (**a**) moved down; (**b**) moved up; (**c**) moved left; (**d**) moved right.

**Figure 23:**
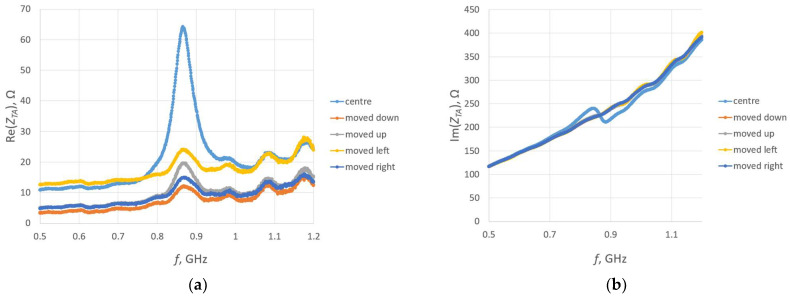
Impedance of transponder antennas *Z_TA_*: (**a**) real part *R_TA_*; (**b**) imaginary part *X_TA_*.

**Figure 24:**
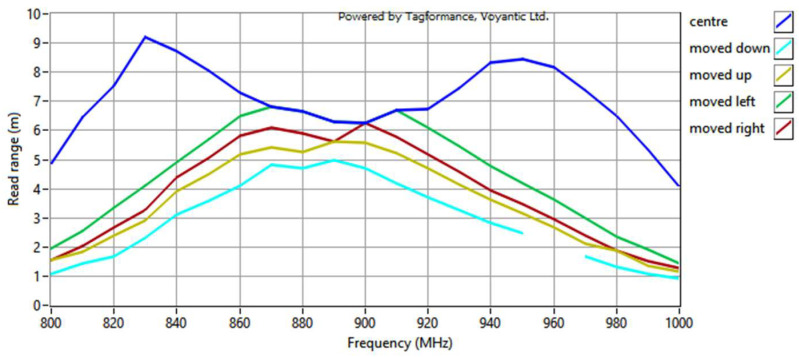
Read range of textronic UHF RFID transponders with different locations of the microelectronic module relative to the antenna coupling circuit.

The same structure of the RFIDtex transponder is examined in this investigation. The four cases of displacement are considered relative to the center of the antenna radiator—shifts downwards, upwards, left, and right (Figure 22). The assumed distance between the centers of the coupling circuits is equal to the length of the loop radius (circa 2.85 mm). For each case, the transponder antenna impedance is measured (Figure 23). Next, the read range for each displacement is obtained (Figure 24).

The obtained results of the read range correspond to the values of the magnetic flux density presented in Figure 8. The meander line antenna is symmetrical, and in this regard, the right and left sides can be interchanged. When simulating the magnetic field distribution, the same results for shifts to the right and left might be expected due to the symmetry of the antenna. However, the direction of the shift also matters in both the computer calculations and the laboratory measurements, which are consistent with each other.

In summary, the results of the study indicate a greater vulnerability to vertical displacement when examining the impact of vertical and horizontal shifts. The same or even lower values were obtained for vertical changes compared to those observed for the experiments in the horizontal direction. However, the maximum vertical distance was 1 mm, whereas the maximum horizontal shift equaled approximately 2.85 mm. This is indeed a disadvantage of the proposed RFIDtex tag design, as distances as small as 1 mm may occur due to even small thread pulls in the antenna embroidery or fabric beneath the microelectronic module. The microelectronic module should also be precisely sewn to the fabric to ensure that the antenna fits tightly to its rigid construction. It can be also observed, based on the analysis of the relationship between the chip voltage *U_TC_* and the reactance *X_M_*, as well as the distribution of the magnetic field, that the transponder with the dipole antenna exhibits less sensitivity to coupling system displacements than the construction with the meander line antenna.

#### 3.2.3. Impact of Antenna Resistance

The influence of the embroidered antenna resistance *R_A_* is elaborated upon in the subsequent investigation. The *R_A_* depends directly on the thread resistivity and the antenna length. The same 16 cm long dipole antenna is used as in the simulation in Section 3.1.2. The same design is embroidered with nine various conductive threads. The antenna resistance is calculated based on the thread resistivity provided by the producers (Table 1).

For each thread, the same antenna was sewn several times. The samples of the PA group were embroidered with a global thread tension of 0 and a tension of 9. For groups PC, PE, PF, PG, PH, and samples PD1-PD3 and PJ1-PJ3, PB1-PB3, the tension was set to 9 and the global tension was set to 8. For samples PD4, PJ4, PB4, and PB5, the global tension was changed to the value of 0.

**Figure 25:**
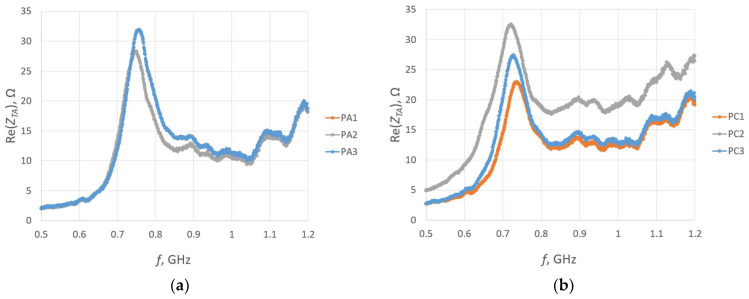

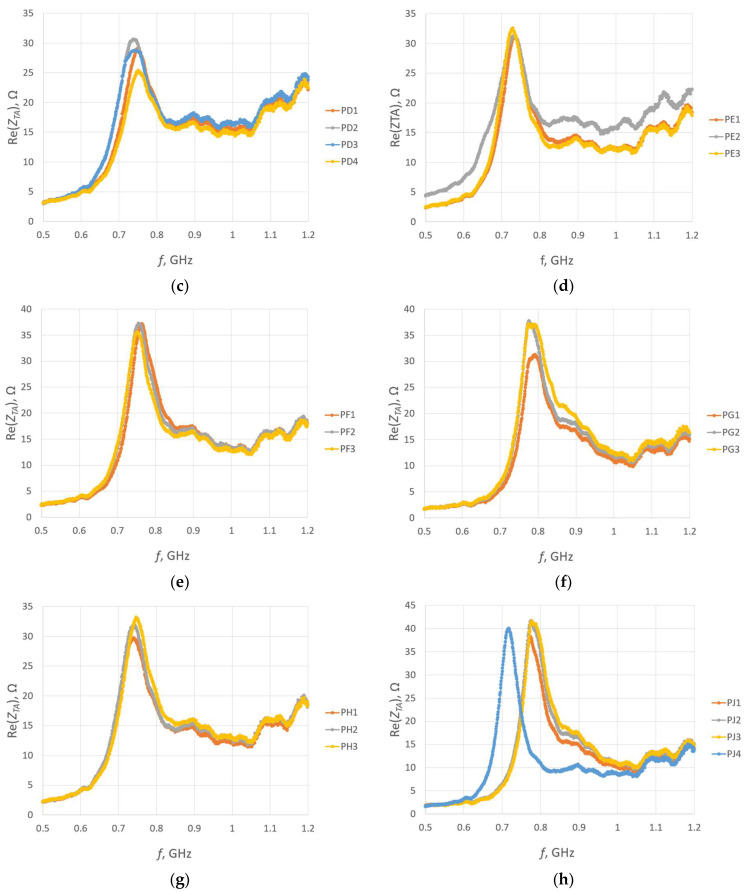
Real part of the transponder antenna impedance for the following groups: (**a**) PA; (**b**) PC; (**c**) PD; (**d**) PE; (**e**) PF; (**f**) PG; (**g**) PH; (**h**) PJ.

**Figure 26:**
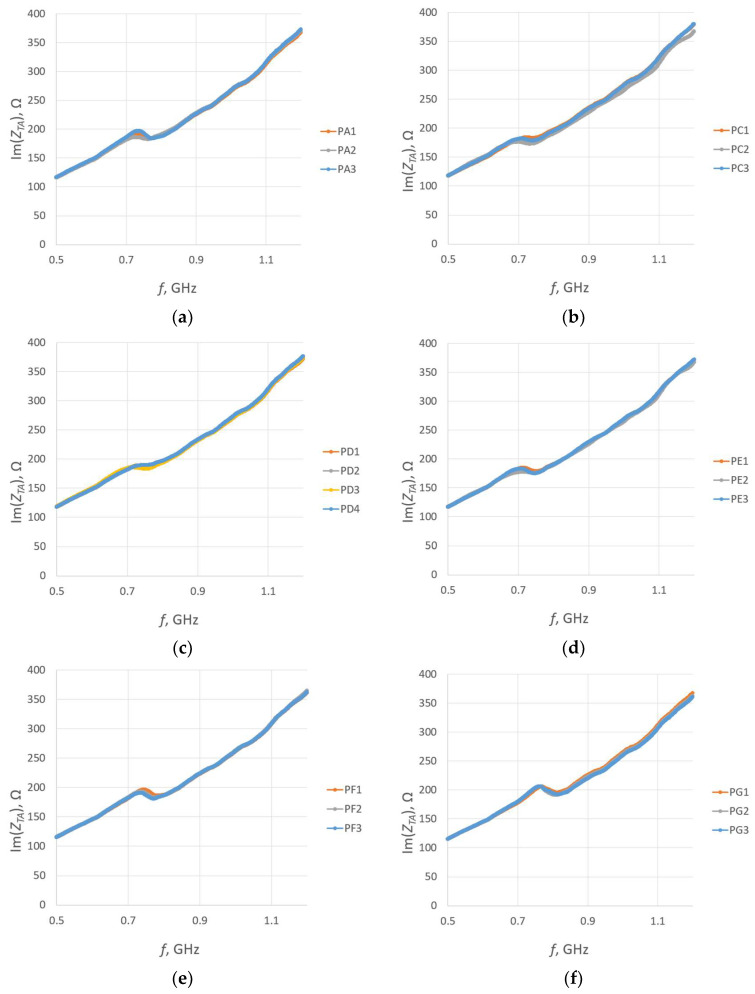

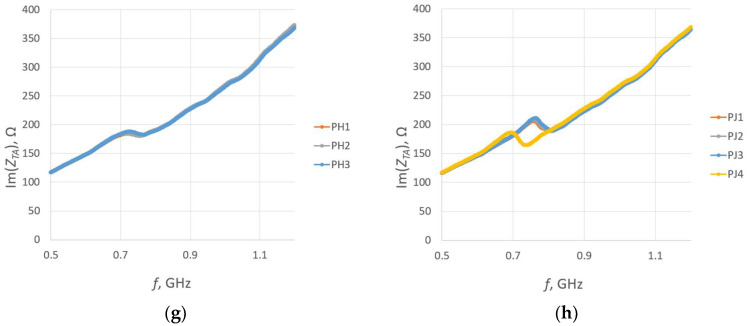
Imaginary part of the transponder antenna impedance for the following groups: (**a**) PA; (**b**) PC; (**c**) PD; (**d**) PE; (**e**) PF; (**f**) PG; (**g**) PH; (**h**) PJ.

For each sample, a microelectronic module without a chip was attached to measure the impedance *Z_TA_* of the transponder antenna. The measurements are presented in Figure 25 and Figure 26.

**Figure 27:**
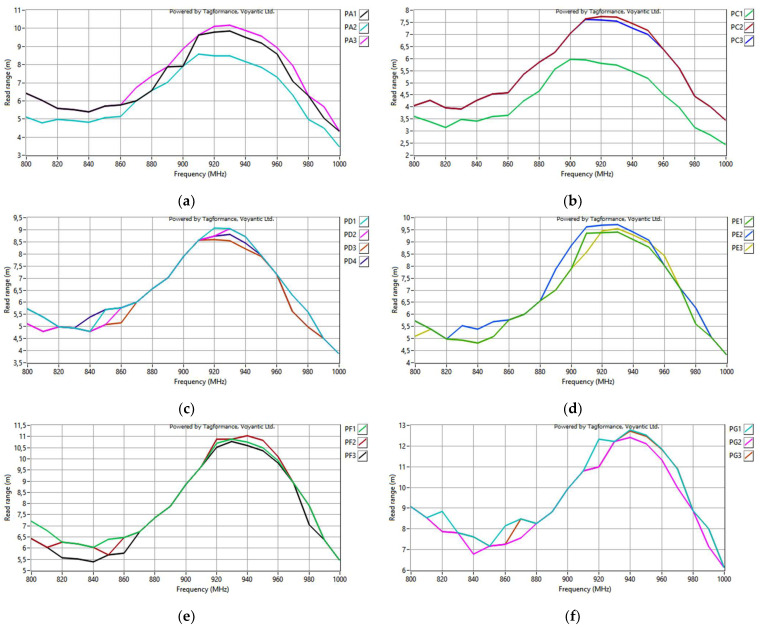

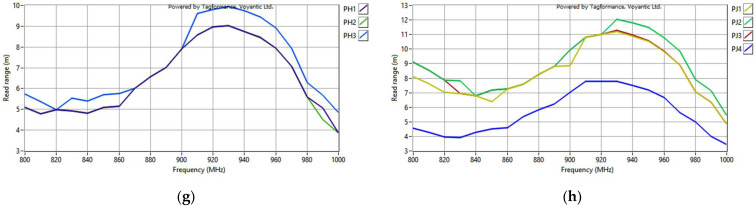
Read range of the transponder antennas for the following groups: (**a**) PA; (**b**) PC; (**c**) PD; (**d**) PE; (**e**) PF; (**f**) PG; (**g**) PH; (**h**) PJ.

In the next step, for each sample, the microelectronic module with the chip is appended, and the read range is measured (Figure 27). The parameters of the measurement process were the same as in the previous study, as described in Section 3.2.1.

The antennas from groups PB and PJ4 were omitted in the analysis of measurements due to failures in embroidering the geometry. The fabricating issues are also presumed to provoke shifts in the resonant frequency. This is described in detail in Section 3.2.4.

The decrease in the impedance in some groups is probably the result of inaccurate alignment in the coupling systems. This impact is presented in Section 3.2.2. The exact alignment is difficult to maintain under laboratory conditions because, during measurements, the microelectronic module is not permanently sewn to the antenna. The microelectronic module is placed on the antenna module, and the alignment with the coupling circuit is performed manually each time.

**Figure 28:**
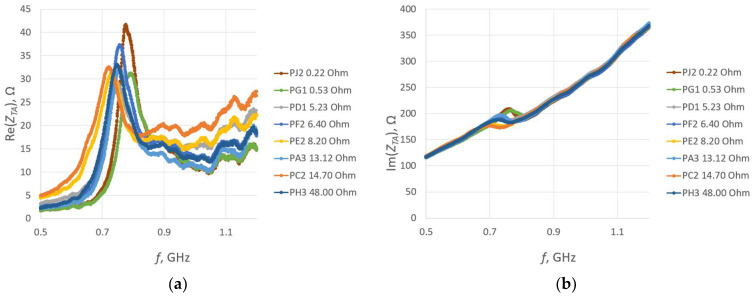
Impedance of transponder antennas sewn using different threads: (**a**) real part; (**b**) imaginary part.

To compare different threads, one sample was chosen from each group (excluding PB) for which, within its respective group, the read range was the largest. A comparison of the impedance *Z_TA_* of these samples is shown in Figure 28.

The obtained results strongly correspond to the graph in Figure 14a. Shifts in the resistance were observed between groups, what is considered to be caused by the manufacturing issues analyzed in Section 3.2.4.

**Figure 29:**
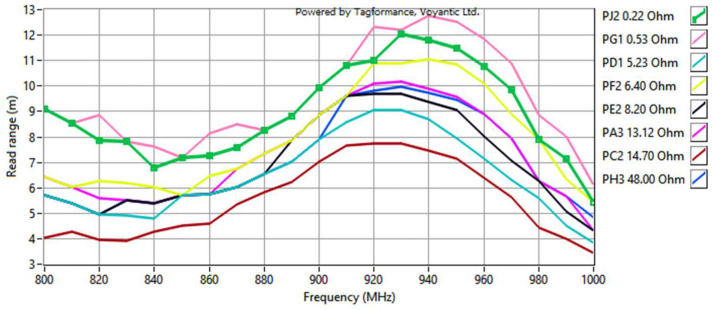
Read range of RFIDtex transponders sewn using different threads.

The comparison of the read range for the samples sewn using various threads is presented in Figure 29.

The accuracy of both the impedance and read range measurements may be influenced by the positioning of the microelectronic system. Conversely, the interpretation of the obtained results may be disrupted due to incorrect data on the thread resistivity provided by the producers. As can be seen from the graphs, the chip voltage of the PD sample was lower than expected when compared to the results for groups with the resistance *R_A_* of the closest value. This may suggest that the resistivity of the thread used in the PD group can be higher than the value specified by the producer.

The read range values reached between the groups correspond with the graph shown in Figure 13a. Nevertheless, the maximum values are shifted towards higher frequencies. The influence of the embroidered antenna resistance is limited to a certain value, which means that above this threshold, there is little to no change in the transponder performance.

#### 3.2.4. Vulnerability to Manufacturing Issues

**Figure 30:**
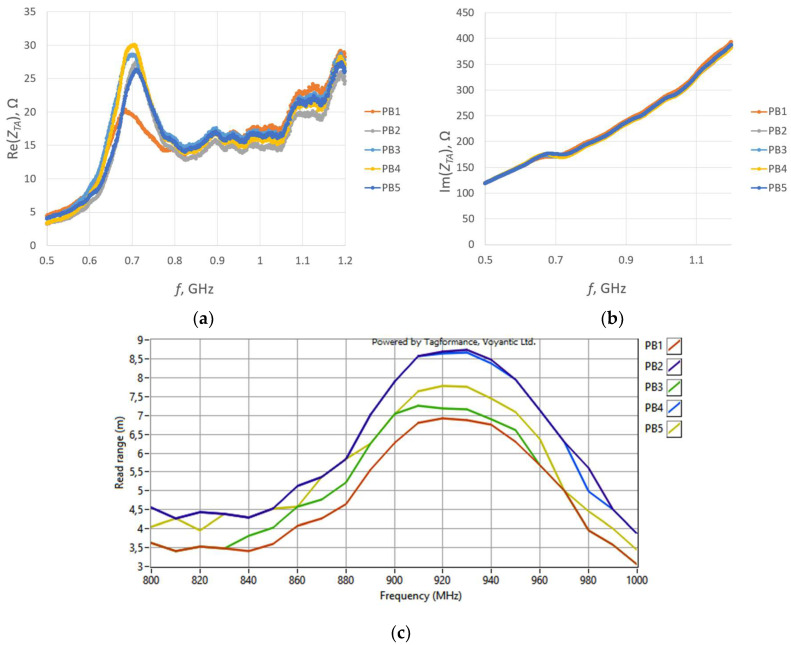
Measurements of group PB: (**a**) real part of antenna impedance; (**b**) imaginary part of antenna impedance; (**c**) read range of textronic UHF RFID transponders.

The samples from the PB group sewn using Adafruit 603 thread are omitted in the analysis of manufacturing issues. The reason for this decision is the discrepancy in the obtained measurements for this sample (Figure 30). This fault indicates poor antenna embroideries, which are caused by technical difficulties in the manufacturing process. Interestingly, no variation in the read range is observed with respect to frequency. This result is surprising, as the other parameters are unstable; however, it is consistent with the expected results based on the relationship between *U_TC_* and *R_A_*, described in Section 3.1.2.

**Figure 31:**
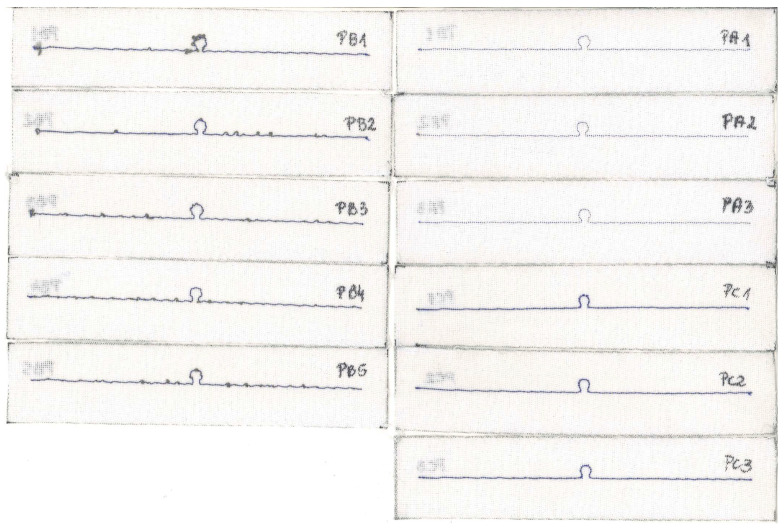
Scans of textronic UHF RFID transponders sewn using different threads; groups PA, PB, and PC.

The reason for the differences between the charts lies in the faulty stitching of the antennas. In Figure 31, the samples for groups PA–PC are presented. The upper thread in the embroidery machine is a regular blue color. It supports the grey conductive thread, which serves as the lower thread in the machine. The visible blue thread knots at the ends of the radiators on the scans do not constitute part of the antenna’s conducting element.

The grey conductive thread in the PB group significantly tangles, making it impossible to stitch the desired shape, both small loops and long straight segments. This is because the thread is too thick in relation to the parameters of the used embroidery machine. Although the maximum read range measurements obtained in this group are higher than the worst results from correctly sewn samples, the discrepancies between the samples in the PB group prevent the use of this thread. The precise and repeated stitching of the same geometry using this model of embroidery machine is not possible.

**Figure 32:**
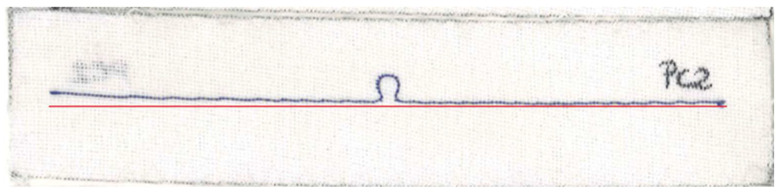
Curvature from the straight line of the left arm of the PC2 antenna.

As can be seen in Figure 25b or Figure 26b, the resonant frequency of sample PC2 is lower than that of the other antennas. The cause of this can also be attributed to the quality of the sample’s fabrication. The right arm of the PC2 antenna is straight, but the left one is clearly curved. In Figure 32, a straight red line is marked, from which the left arm of the antenna deviates.

**Figure 33:**
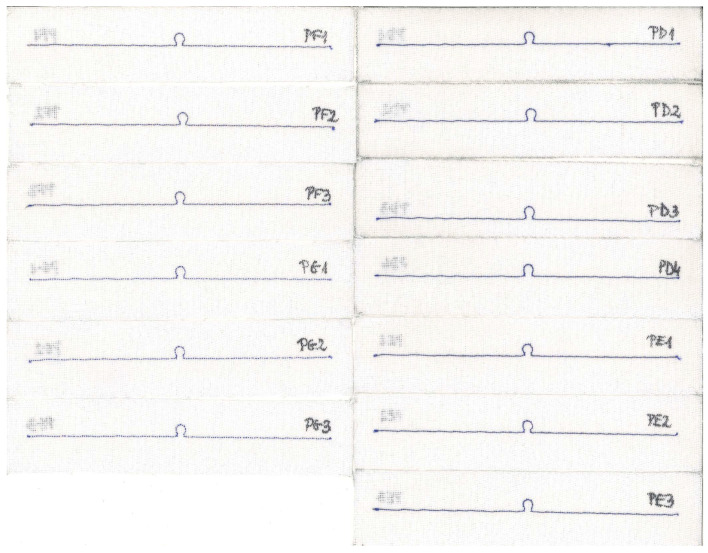
Scans of textronic UHF RFID transponders sewn using different threads; groups PD, PE, PF, and PG.

The samples of the antennas from groups PD–PG are shown in Figure 33. It can be observed in Figure 25c that the PD2 and PD3 antennas have a lower resonant frequency than the others. As seen in the scans, the left arm of these antennas is noticeably wavier compared to samples PD1 and PD4.

**Figure 34:**
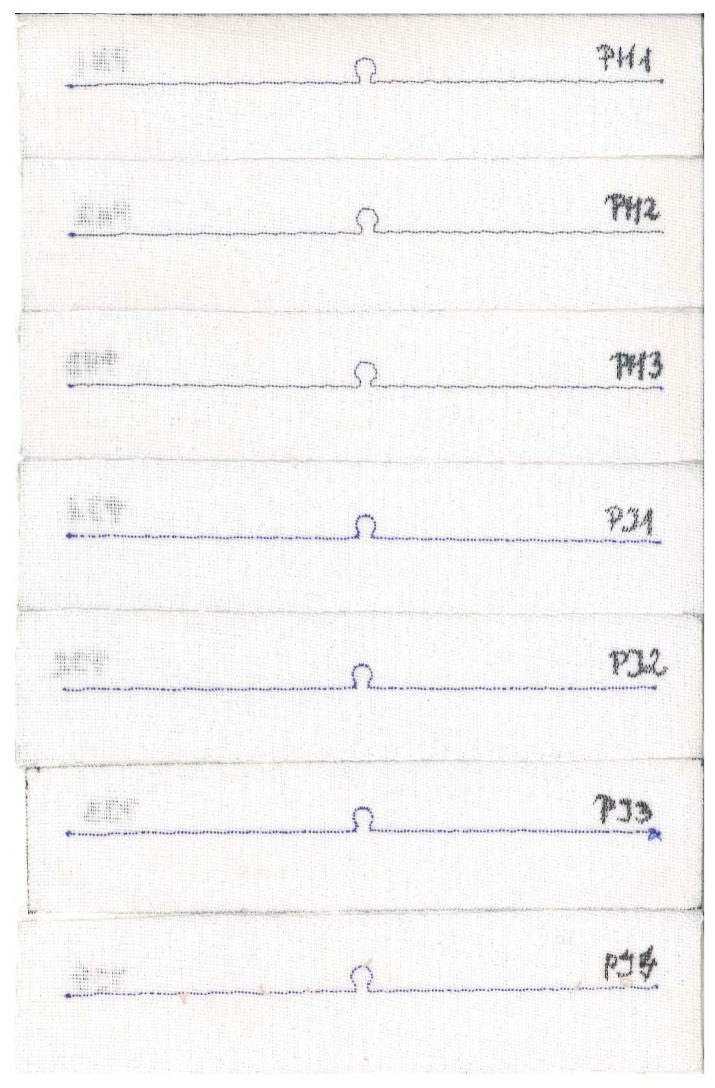
Scans of textronic UHF RFID transponders sewn using different threads; groups PH and PJ.

The last two groups of RFIDtex tags are presented in Figure 34. As seen from the graphs in Figure 25h and Figure 26h, the measurements for sample PJ4 significantly differ from the others.

In the PJ4 antenna, grey loops of conductive thread protruding from the embroidery can be seen. These result from an attempt to embroider the antenna using a conductive thread that has an excessively high stiffness.

## 4. Discussion

The significant dependence on the distance between the centers of coupling coils is concerning, because even small deviations prompt substantial changes in the operational parameters. These deviations can be caused, for example, by the loosening of threads around the sewn button (microelectronic module), its displacement, threads under the button, or material wrinkling. Although the product is manufactured correctly, if the RFIDtex tag is intended to be used throughout the product’s lifecycle, such damage is highly probable to occur over time. Based on numerical calculations, it is presumed that the high sensitivity to such displacements is inherent in construction with the meander line antenna. Applying a different geometry of the antenna module should overcome this problem, which will be investigated in the future.

The conducted research shed light onto an interesting feature of the textronic UHF RFID transponder, i.e., its dependency on the embroidered antenna resistance. A lower thread resistivity results in a higher chip voltage and transponder antenna impedance. However, there is a specific resistivity value for the thread beyond which its variations do not significantly impact the change in the impedance or the induced voltage at the chip terminals. In RFID systems where smaller ranges are sufficient, the resistivity ceases to be crucial in selecting an appropriate thread (or another material suitable to manufacture a textile antenna). This is especially useful, considering the challenges in measuring thread resistivity and the possibility of incomplete or inaccurate information provided by manufacturers. This feature also indicates that a deterioration in thread resistivity during product usage may not alter the transponder’s operational parameters. And in the case of low resistivity threads, it might be possible to estimate such changes. However, it should be emphasized that this conclusion is correct under the assumption that product usage will not adversely affect other thread characteristics such as changes in the antenna geometry or mechanical injuries.

In light of the above considerations, when designing an RFIDtex transponder, a greater challenge than selecting a thread with an appropriate conductivity or monitoring its changes over time is ensuring accuracy in the fabrication of the antennas. Even minor thread pulls or stitch curvatures caused significant deviations from the expected results. Therefore, in systems where threads with lower conductivities provide sufficient transponder performance, the chosen thread should be selected for ease of use in embroidering the correct geometry and ensuring repeatability of measurements for subsequent samples.

The next step in the identification of factors affecting the performance of the textronic UHF RFID transponder was the exploration of various geometries of antenna radiators and coupling circuits. At this stage of the research, significant differences in the operation of transponders were observed solely due to the application of different antenna geometries in their structures. The strong influence of geometry on the transponder’s efficiency is further evidenced by issues arising from incorrect stitching or imprecise placement of the microelectronic circuit in relation to the antenna. Thus, different dimensions and shapes of coupling systems should be taken into account. It will be necessary to develop methods that allow for the selection of coupling system dimensions so that, for given antenna and module shapes, it will be possible to approach the maximum of the chip voltage. For the developed microelectronic module designs, corresponding forms of clothing elements and the most convenient locations for their application will be also proposed. The drawn conclusions will contribute to the future development of rules concerning the design and implementation of structures that fulfil the idea of the textronic UHF RFID transponder and the collection of tools that are useful in this process.

Other research directions include the following: determining the impact of environmental factors on the transponder’s operation arising from its proximity to the human body such as temperature or humidity; mechanical durability against interactions occurring during clothing usage, cleaning, and maintenance; or effectiveness during moments when such interactions occur, e.g., garment bending. The aforementioned studies would allow for a determination of the usefulness and applicability of the textronic UHF RFID transponder for RFID systems accompanying the user in everyday situations after the product purchase.

## 5. Conclusions

This article discusses the impact of the following factors on the textronic UHF RFID transponder’s operation: system geometry, mutual inductance in coupling systems, displacement between the coupling circuits, and the resistance of the embroidered antenna. Displacements, both in vertical and horizontal directions, strongly affect the impedance of the transponder antenna and the transponder read range. A greater sensitivity to coupling circuit displacement in the vertical direction than in the horizontal direction was observed. This impact is presumed to be weaker for the dipole antenna, which will be verified in future studies. A small or no influence of the embroidered antenna resistance on the transponder antenna impedance and the transponder read range was detected, what means that above a certain value of the thread resistivity, no deterioration in the transponder’s performance parameters is observed.

## Figures and Tables

**Table 1:** Resistivity of conductive threads and resistance of embroidered 16 cm long dipole antenna.

Group Name	Thread	Thread Resistivity, Ω/m	Antenna Resistance *R_A_*, Ω
PA	AGSIS SYSCOM	82.00	13.12
PB	ADAFRUIT 603	39.37	6.30
PC	SPARKFUN DEV-11791	91.86	14.70
PD	ADAFRUIT 641	32.68	5.23
PE	ADAFRUIT 640	51.18	8.19
PF	ELECTRO FASHION	40.00	6.40
PG	LIBERATOR 40	3.28	0.52
PH	INNTEX PW018A	300	48.00
PJ	LICA 10 × 0.04 mm	1.39	0.22

## Data Availability

All the calculated and measured data will be provided upon request to the correspondent authors via email with an appropriate justification.
